# Seroprevalence and assessment of public awareness of *Brucella* spp., *Toxoplasma gondii* and *Chlamydia abortus* in small ruminants from selected smallholder commercial farms of Zimbabwe

**DOI:** 10.1371/journal.pone.0287902

**Published:** 2023-06-29

**Authors:** Dey F. Tarusikirwa, Barbara Blacklaws, Caroline L. Trotter

**Affiliations:** Department of Veterinary Medicine, University of Cambridge, Cambridge, United Kingdom; Beni Suef University Faculty of Veterinary Medicine, EGYPT

## Abstract

*Brucella* spp., *Toxoplasma gondii*, and *Chlamydia abortus* have long been recognized as zoonoses and significant causes of reproductive failure in small ruminants globally. A cross-sectional study was conducted in August 2020 to determine the seroprevalences of *Brucella* spp., *Toxoplasma gondii* and *Chlamydia abortus* in 398 small ruminants from four districts of Zimbabwe (Chivi, Makoni, Zvimba, and Goromonzi) using Indirect-ELISAs. A structured questionnaire was used to assess the knowledge, attitudes, and practices of 103 smallholder farmers towards small ruminant abortions, *Brucella* spp., *T*. *gondii* and *C*. *abortus*, and to obtain a general overview of the significance of small ruminant reproductive failure(s) on their livelihoods. The overall seroprevalences were: 9.1% (95% CI: 6.4–12.3) for *Brucella* spp., 6.8% (95% CI: 4.5–9.7) for *T*. *gondii* and 2.0% (95% CI: 0.9–3.9) for *C*. *abortus*. Location, age, parity, and abortion history were associated with *Brucella* spp. seropositivity. Location was also associated with both *T*. *gondii* and *C*. *abortus* seropositivity. The questionnaire survey established that 44% of respondents had recently faced reproductive disease challenges within their flocks, with 34% correctly identifying abortion causes and only 10%, 6% and 4% having specific knowledge of *Brucella* spp., *C*. *abortus* and *T*. *gondii*, respectively. This study provides the first serological evidence of *Brucella* spp. in small ruminants since 1996 and builds the evidence on small ruminant toxoplasmosis and chlamydiosis in Zimbabwe. Evidence of these zoonoses in small ruminants and the paucity of knowledge shows the need for a coordinated One Health approach to increase public awareness of these diseases, and to establish effective surveillance and control measures. Further studies are required to establish the role these diseases play in small ruminant reproductive failure(s), to identify the *Brucella* spp. detected here to species/subspecies level, and to assess the socio-economic impact of reproductive failure in livestock among marginalised rural communities.

## Introduction

The livelihoods of smallholder commercial farmers in Zimbabwean communal areas are dependent heavily on small ruminant production [1–4]. Small ruminants are mainly used for food and nutritional security, income generation, capital storage, manure, as a status symbol, and in important socio-cultural activities and rituals [1–3, 5, 6]. Furthermore, they also utilize idling resources like crop residues that would probably go to waste [2, 4]. However, small ruminant production faces a myriad of challenges which include diseases; inadequate grazing and browsing grounds; poor feed supplementation; poor husbandry practices and lack of adequate veterinary care [2–5, 7, 8]. Among the disease problems, abortions have proven to be a particular challenge causing considerable economic losses to smallholder farmers, through the loss of replacement and income generating stock(s), increased lambing/kidding intervals, increased culling rates and decreased breeding stock value [4, 9–12]. Thus, small ruminant production related benefits can only be maximised if the causes of reproductive failure such as abortions are identified and controlled. Moreover, abortions may be of public health importance if they are induced by zoonoses, as women and children play an active role in small ruminant husbandry in African rural areas [6, 13]. Thus, if women of childbearing age are exposed to small ruminants harbouring these infections the effects may be detrimental.

Currently in Zimbabwe, there is little documentation on the epidemiology and surveillance of zoonotic abortion causing agents in small ruminants. However, *Toxoplasma gondii*, *Brucella* spp. and *Chlamydia abortus* have long been recognized as zoonoses, and causes of abortion storms, stillbirths, and neonatal mortalities in small ruminants [11, 14, 15]. Previous studies revealed that farmers in Zimbabwe had little knowledge on the transmission and risk factors of zoonotic causes of abortions in livestock [12, 16, 17]. This lack of awareness of zoonoses coupled with poverty, especially in rural communities, results in many people accessing unpasteurised fresh milk and uninspected meat from informal food markets [16, 18]. This puts people at risk of infection, as these pathogens easily spread from animals to humans [11, 18]. In Zimbabwe, most causes of reproductive failure such as abortions in small ruminants remain unexplored and their link with the seroprevalence(s) of brucellosis, toxoplasmosis, and chlamydiosis have not been investigated. Owing to the high human dependence on animals and animal products for livelihoods in rural communities, it was imperative to investigate small ruminant brucellosis, toxoplasmosis, and chlamydiosis. We determined their seroprevalence(s) using serological tests; and assessed the knowledge, attitudes, and practices smallholder farmers have towards these diseases, small ruminant husbandry and reproductive failure; as well as obtaining a general overview of the significance of small ruminant reproductive failures on the livelihoods of these farmers, in order to assess their public health significance.

## Materials and methods

### Study setting

According to the Department of Livestock and Veterinary Services, the Central Statistics Office livestock census of 2019 indicated that Zimbabwe had an overall small ruminant population of 4,625,066 (goat = 2,770,294; sheep = 1,854,772) (refer to S1 Table). Small ruminant husbandry is mainly practised in communal land areas in the drier regions of Zimbabwe (Agroecological regions III to V), which are characterised by erratic and low annual rainfall (below 500–650 mm) and poor pastures [4, 5, 19]. In these regions goats, are highly valuable assets for food security, income generation and capital investment [4], as crop production is semi-extensive [5, 19]. However, it is not limited to these regions as smallholder farmers in the wetter agroecological regions (I to IIb) (annual rainfall >700 mm) also practise it, for both subsistence and income generation [4, 5]. In all regions of Zimbabwe small ruminant husbandry mainly relies on the communal free-range grazing system for nutrition where animals are kept in pens overnight and share state-owned communal grazing and browsing grounds during the day [4, 5]. This encourages inter-flock mixing in communal pastures, which could potentially lead to disease spread from infected to susceptible animals [17, 20, 21].

**Fig 1:**
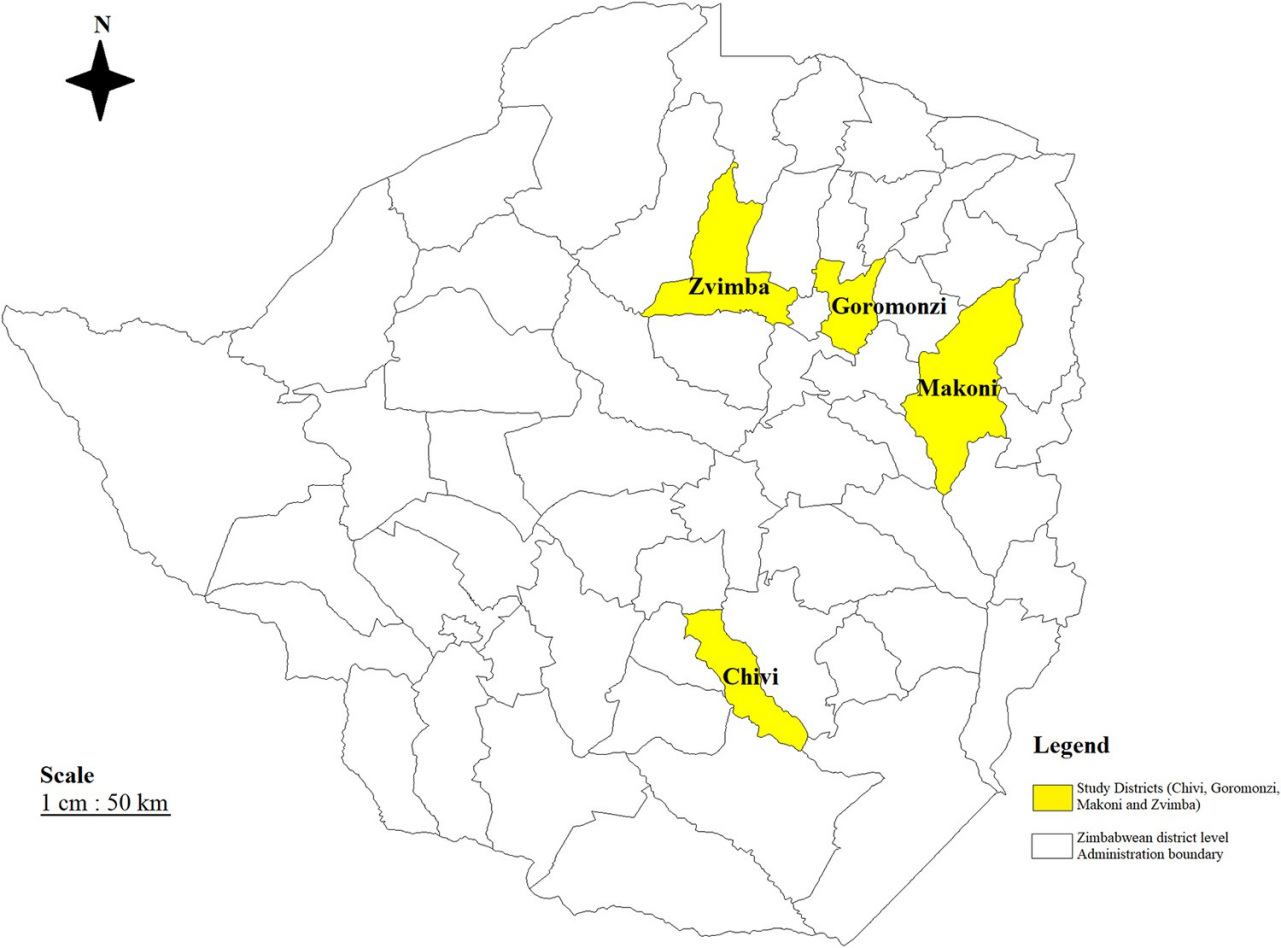
Map of Zimbabwe showing the sampled districts. (This map is our own developed from Zimbabwe shape files using DIVA-GIS Software (version 7.5). The coloured regions represent the districts in which communal land areas were sampled).

This study was performed in communal land areas from three districts: Chivi, Makoni, and Zvimba, and at a grade B abattoir in Goromonzi district (Fig 1). By definition smallholder farmers are farmers who are located in communal and resettlement small-scale farming areas with landholding of up to 35 hectares [22]. Chivi district is a semi-arid to arid area, with areas that fall in agroecological region IV and V [23]. Chivi is a rural settlement that is characterised by the clustering of households, limited farming lands and communal grazing lands for livestock. Makoni district is in agroecological region IIb, while both Zvimba and Goromonzi districts are in agroecological region IIa [23]. Communal areas in these 3 districts are characterised by intensive crop production, and limited land use for livestock pastures [4, 5, 23], households tend to be less clustered, but because of limited land for both farming and pastures, farmers also practise communal grazing. It is worth noting that the abattoir slaughters small ruminants from both smallholder and large-scale commercial farms from its surrounding areas.

### Study design

A cross-sectional study was conducted from 10–23 August 2020 to determine the seroprevalence of *Brucella* spp., *Toxoplasma gondii*, and *Chlamydia abortus* in adult sheep and goats (≥1 year old) from four districts in Zimbabwe; to assess the knowledge, attitudes, and practices (KAP) of smallholder farmers towards brucellosis, toxoplasmosis, chlamydiosis, small ruminant husbandry and reproductive failures; and to obtain a general overview of the significance/impact of small ruminant reproductive failure on the livelihoods of smallholder farmers. After communal areas were identified through review of small ruminant population sizes (refer to S1 & S2 Tables), agroecological regions and liaison with local government veterinary personnel, the three districts (Chivi, Makoni, and Zvimba) were selected based on the availability of resources, and ease of both access to the geographical locations and logistics. The final sampling site a Grade B abattoir (Goromonzi district) was selected for comparison between husbandry practices (extensive vs semi-intensive/intensive) and slaughter types (informal vs formal).

A dip tank is a functional unit used for livestock disease control and surveillance activities in Zimbabwean communal areas [19]. The local government veterinary services office within that area keeps a database of all farmers who are likely to use any of the dip tanks [17]. As such a farmer from the database was taken to represent a household. For goat/sheep blood collection, farmers from the three districts were conveniently selected based on their willingness to participate in the study. After the households and abattoir were selected, the sheep/goats were identified and sampled using a simple random method. Baseline data of the animals (age, parity, sex, and reproduction health history) were collected using a sampling chart from the owners/animal attendants during sampling. For the questionnaire survey, voluntary participants were randomly selected from the database.

### Sample size determination

We used a simple random sampling formula as previously described [24] and expected prevalence(s) of 67.2% for *T*. *gondii*, 22% for *C*. *abortus* [5, 19], and 50% for *Brucella* spp. [24] with 5% desired absolute precision at 95% confidence level to estimate the desired sample size. A minimum overall sample size of 339 for *T*. *gondii*, 264 for *C*. *abortus*, and 385 for *Brucella* spp. were required. These minimum target sample sizes were reached by sampling a total of 398 small ruminants by the end of the study. Overall, 335 goats [Chivi district (n = 138 from 12 flocks), Zvimba district (n = 124 from 6 flocks), Makoni district (n = 39 from 2 flocks), and Goromonzi district (n = 34 from Grade B abattoir)], and 63 sheep [Zvimba district (n = 38 from 2 flocks) and Makoni district (n = 25 from 2 flocks)] were sampled. Because most of the sampled animals were from communal land areas, the sample size was not adjusted for clustering by flock.

### Data collection

#### Sample collection

After aseptic skin preparation, blood samples (4 ml) were collected by jugular venepuncture into plain vacutainer tubes (Vacucare, EREZlabmed, South Africa), identified, and sent to Diagnopath Medical Laboratory, Harare, Zimbabwe under chilled conditions (between 2–8°C). On arrival, clotted blood samples were centrifuged (Hettich Rotofix 32A benchtop centrifuge, Massachusetts, USA) at 1400 x ***g*** for 5 minutes to obtain sera. Sera were then placed in 2 ml screw-on Eppendorf vials (Microcentrifuge tubes, EREZlabmed, South Africa), identified, and stored at -20°C until the time of shipment to the University of Cambridge. The samples were then packaged according to IATA regulations and were transported on dry ice (-80°C) with temperature monitoring from Diagnopath Medical Laboratory, Harare, Zimbabwe to the Department of Veterinary Medicine, University of Cambridge, Cambridge, United Kingdom. On arrival the samples were unpacked and stored at -20°C until the time of testing.

#### Questionnaire survey

A total of 103 (Chivi (n = 37), Zvimba (n = 35), and Makoni (n = 31)) structured questionnaires with both closed- and open-ended questions were administered by the end of the study. The questionnaire was informally pre-tested on six veterinary professionals prior to sampling. The defects and ambiguity of the questions were noted and revised to improve the accuracy of the data collected. The questionnaire was administered by the principal investigator and a trained assistant to the household head, spouse, or animal attendant (≥18 years), and interviews lasted between 20–25 minutes each. The questionnaire was administered in English and where necessary for clarity was translated into the local language (Shona language). The questionnaire captured data on household and livestock ownership demographics, small ruminant husbandry practices and health-related issues including knowledge on causes of reproductive failures and their transmission pathways. Furthermore, it also captured information on the knowledge and attitudes toward zoonoses particularly brucellosis, toxoplasmosis, and chlamydiosis, and issues relating to risky animal husbandry practices that expose farmers to these zoonoses. It also tentatively captured information on the uses of small ruminants and the magnitude of reproduction health related issues on their production.

#### Laboratory tests

For anti-*Brucella* spp. antibody screening the ID Screen® Brucellosis Serum Indirect Multi-species ELISA (ID.vet, Montpellier, France) was used. It is an indirect enzyme linked immunosorbent assay (iELISA) which detects anti-*Brucella* spp. IgG antibodies against *B*. *abortus* LPS in serum and plasma. For anti-*T*. *gondii* antibody screening the ID Screen® Toxoplasmosis Indirect Multi-species ELISA (ID.vet, Montpellier, France) was used. It is an iELISA which detects anti-*T*. *gondii* IgG antibodies against *T*. *gondii* P30 antigen in sera, plasma, and meat juices. For anti-*C*. *abortus* antibody screening the ID Screen® *Chlamydophila abortus* Indirect Multi-species ELISA (ID.vet, Montpellier, France) was used. It is an iELISA which detects anti-*C*. *abortus* IgG antibodies against a synthetic antigen from a major outer-membrane protein (MOMP) specific to *C*. *abortus* in serum or plasma.

#### Testing procedure, results interpretation and validation

For all the three serological assays, testing and the interpretation and validation of results were conducted according to the manufacturer’s recommendations (refer to: S5–S7 Files).

### Statistical analysis

The data generated from the serological survey was captured and coded using a Microsoft Excel spreadsheet (Microsoft Corporation). Epi Info^TM^ version 7.2.4.0 (Centres for Disease Control and Prevention, USA) and a Microsoft Excel spreadsheet (Microsoft Corporation) were used to capture and store the questionnaire survey data respectively. For all data, descriptive and analytic statistics were performed using standard functions in STATA® version SE 16.1 for Windows (Stata Corp, College Station, Texas, USA).

The seroprevalences were determined using the total number of small ruminants that had a positive iELISA result over the total number of animals tested for *Brucella* spp., *T*. *gondii*, and *C*. *abortus*, respectively. The test prevalence (P) estimates were then adjusted to account for the test sensitivities and specificities and the adjusted prevalence(s) (adj.P) were determined as previously described [24]. Flock I.D./size was not considered as a predictor variable because of the communal sharing of pastures in these regions. Here animals mix freely between flocks, with some farmers encouraging this practise for interbreeding purposes [4, 17]. This may provide an opportunity for the uniform spread of diseases like brucellosis and chlamydiosis following massive environmental contamination from abortion or parturition when infected does/ewes shed copious amounts of infective material, thereby confounding the effect(s) of flock I.D./size on seroprevalence [17, 19, 25–27]. Using Pearson’s chi-squared and Fisher’s exact tests (for comparison), two-way tables with measures of association were used to test for association between seropositivity and each predictor variable for each disease, respectively. Predictor variables that showed a significant association (p<0.05) in Pearson’s chi-squared and Fisher’s exact tests were included in the univariable logistic regression analysis. For *Brucella* spp. predictor variables that had a significant association (p<0.05) in the univariable analysis were included in the multivariable logistic regression model. Odds ratios and their 95% confidence intervals were used to evaluate the strength of association between seropositivity and the various predictor variables. The questionnaire survey data analysis was focused on the generation of descriptive statistics (frequency distributions) on the collected responses.

### Ethics approval

Ethical approval for the use of small ruminants and all protocols used in this study was granted by the National Animal Research Ethics Committee (NAREC), Division of Veterinary Technical Services, Department of Livestock and Veterinary Services, Ministry of Lands, Agriculture, Water, Climate and Rural Resettlement, Zimbabwe (Reference Number: NAREC/006/2020, Approval date: 16/07/2020). Permission to conduct the study was also granted by the University Biomedical Services (UBS), Animal Welfare and Ethical Review Board (AWERB), University of Cambridge (AWERB meeting: 29/07/2020). The purpose of the study was fully explained to each of the participants, and verbal consent was obtained before the participants took part in either or both aspect(s) of the study, that is the small ruminant serosurvey and the questionnaire survey. The data obtained from each participant during the study was kept confidential.

### Biological material movement approval

Import approval/licence for the shipment of small ruminant sera from Zimbabwe into the United Kingdom was obtained from the Animal and Plant Health Agency, Department of Environment, Food and Rural Affairs, United Kingdom (Authorisation Number: ITIMP20.1150, Approval date: 10/11/2020). The export approval/licence for the shipment of small ruminant sera from Zimbabwe into the United Kingdom was obtained from the Department of Livestock and Veterinary Services, Ministry of Lands, Agriculture, Water, Climate and Rural Resettlement, Zimbabwe (Reference Number: VB/2/13, Approval date: 26/01/2021).

### Inclusivity in global research

Additional information regarding the ethical, cultural, and scientific considerations specific to inclusivity in global research is included in the S8 File).

## Results

### Serosurvey: *Brucella* spp.

The overall unadjusted seroprevalence for brucellosis was 9.1% (95% CI 6.4–12.3%) and after adjustment was 9.2%. Location showed a significant association with seropositivity (Fisher’s exact, p<0.0001), with seropositivity being greatest in animals from the abattoir (Goromonzi) (76.5%), compared to Makoni (15.6%), while Chivi and Zvimba did not record any seroreactors. Age also showed a significant association with seropositivity (Fisher’s exact, p<0.0001), however, no animals <2 years were seropositive and animals >5 years recorded the highest seropositivity (22.8%). Both parity (Fisher’s exact, p = 0.01) and abortion history (Fisher’s exact, p = 0.002) also showed a significant association with seropositivity.

The univariable logistic regression analysis indicated that animals from the abattoir (Goromonzi district) were 17.6 times more likely to be seropositive for *Brucella* spp. infection compared to Makoni district (OR = 17.6, 95% CI 6.2–49.7, p = 0.000). Older animals (>5 years) had greater odds of being seropositive for *Brucella* spp. infection compared to younger animals (OR = 7.3, 95% CI 2.5–21.6, p = 0.000). Increase in parity (parity ≥3) showed higher odds of *Brucella* spp. seropositivity (OR = 2.9, 95% CI 1.4–6.0, p = 0.005), and animals with abortion history were 3.1 times more likely to be *Brucella* spp. seropositive (OR = 3.1, 95% CI 1.5–6.1, p = 0.002). Based on a stepwise backward elimination procedure, predictor variables that showed a significant independent association (p<0.05) in the univariable logistic regression analyses were included in a multivariable logistic regression model (location, age, parity, and abortion history). The multivariable logistic regression model showed that animals from the abattoir (Goromonzi district) were 25.6 times more likely to be seropositive for *Brucella* spp. infection compared to Makoni district (OR = 25.6, 95% CI 7.4–89.2, p = 0.000), while age, parity, and abortion history did not show any significant associations with seropositivity (p>0.05). See Table 1 for the summarised results.

**Table 1:** Summary of descriptive and analytic statistics for *Brucella* spp. seropositivity in small ruminants from selected smallholder farming areas of Zimbabwe (2020).

Variable	Level	Distribution ^d^			Association testing^d^	Univariable logistic regression^d^		
		*N*	*Prevalence (%)*	*CI (95%)*	*Fisher’s exact*	*OR*	*CI (95%)*	*p value*
Seropositivity	Overall	398	36 (9.1) [9.2]	6.4–12.3	-	-	-	-
Location*^a^	1: Chivi^**o**^	138	0	0–2.6	p<0.000		(empty)	
	2: Zvimba^**o**^	162	0	0–2.3			(empty)	
	3: Makoni	64	10 (15.6)	7.8–26.9		Ref	-	-
	4: Abattoir	34	26 (76.5)	58.8–89.3		17.6	6.2–49.7	0.000
Species	1: Caprine	335	33 (9.9)	6.9–13.6	p = 0.239			
	2: Ovine	63	3 (4.8)	1.0–13.3				
Age*^a^	1: 1<x≤2 years^**c**^	69	0	-	p<0.000			
	2: 2<x≤3 years^**c**^	59	5 (8.5)	2.8–18.7		Ref	-	-
	3: 3<x≤4 years	93	9 (9.7)	4.5–17.6		2.6	0.9–8.1	0.092
	4: 4<x≤5 years	120	9 (7.5)	3.5–13.8		2.0	0.7–6.1	0.228
	5: x>5 years	57	13 (22.8)	12.7–35.8		7.3	2.5–21.6	0.000
Parity*^a^	0: 0^**o**^	1	0	-	p = 0.010		(empty)	
	1: 1–2	212	11 (5.2)	2.6–9.1		Ref	-	-
	2: 3–4^**c**^	144	21 (14.6)	9.3–21.4		2.9	1.4–6.0	0.005
	3: ≥5^**c**^	28	4 (14.3)	4.0–32.7				
Sex	1: Female	385	36 (9.4)	6.6–12.7	p = 0.618			
	2: Male	13	0	-				
Abortion history*^a^	0: No	281	18 (6.4)	3.8–9.9	p = 0.002	Ref	-	-
	1: Yes	104	18 (17.3)	10.6–26.0		3.1	1.5–6.1	0.002

***N*,** number of animals; ***P***, probability value; ***CI***, Confidence Interval

*These variables had Fisher’s Exact *p* < 0.05 and were used in the univariable logistic regression analysis

^**a**^ These variables showed a significant independent association (p<0.05) in the univariable logistic regression analysis and were used in the multivariable logistic regression model

^**d**^ Dependent variable: small ruminant seropositivity for *Brucella* sp. (negative = 0, positive = 1)

^**c**^ These groups were combined to form a single group within that particular level; ^**o**^/empty: These groups were omitted from the regression analyses because they did not have any positives; [%] test adjusted prevalence.

### Serosurvey: *Toxoplasma gondii* and *Chlamydia abortus*

The overall unadjusted seroprevalences for toxoplasmosis and chlamydiosis were 6.8% (95% CI 4.5–9.7%) and 2.0% (95% CI 0.9–3.9%) respectively, and after adjustment they were 5.2% for toxoplasmosis and 0.6% for chlamydiosis. Location was the only predictor variable that showed a significant association (Fisher’s exact, p<0.05) with both *T*. *gondii* and *C*. *abortus* seropositivity. *T*. *gondii* seropositivity per district was 13.8% in Chivi, 11.8% at the abattoir (Goromonzi), 1.9% in Zvimba, and 1.6% in Makoni. While for *C*. *abortus* seropositivity, only Chivi (5.1%) and the abattoir (Goromonzi) (2.9%) recorded seroreactors.

The results of the univariable logistic regression analysis for *T*. *gondii* showed that location had a significant independent association (p<0.05) with seropositivity, with animals from both Makoni (OR = 0.1, 95% CI 0.0–0.8, p = 0.026) and Zvimba (OR = 0.1, 95% CI 0.0–0.4, p = 0.001) districts less likely to be seropositive for *T*. *gondii* infection compared to Chivi district. Age did not show a significant independent association with seropositivity in the univariable analysis, however, being a known confounder, it was included in the multivariable logistic regression model for *T*. *gondii* together with location. The results were similar to those of the univariable analysis, with animals from both Zvimba (OR = 0.1, 95% CI 0.0–0.4, p = 0.001) and Makoni (OR = 0.1, 95% CI 0.0–0.8, p = 0.027) districts less likely to be seropositive for *T*. *gondii* infection compared to Chivi district, while age did not show a significant independent association with seropositivity (2<x≤3 years: OR = 0.7, 95% CI 0.1–3.1, p = 0.61; 3<x≤4 years: OR = 0.7, 95% CI 0.2–2.5, p = 0.55; 4<x≤5 years: OR = 1.0, 95% CI 0.3–3.2, p = 0.98; and x>5 years: OR = 1.0, 95% CI 0.2–4.4, p = 0.99). See Tables 2 and 3 for the summarised results.

**Table 2:** Summary of descriptive and analytic statistics for *T*. *gondii* seropositivity in small ruminants from selected smallholder farming areas of Zimbabwe (2020).

Variable	Level	Distribution ^d^			Association testing ^d^	Univariable logistic regression ^d^		
		*N*	*Prevalence (%)*	*CI (95%)*	*Fisher’s exact*	*OR*	*CI (95%)*	*p value*
Seropositivity	Overall	398	27 (6.8) [5.2]	4.5–9.7	-	-	-	-
Location*	1: Chivi	138	19 (13.8)	8.5–20.7	p < 0.000	Ref	-	-
	2: Zvimba	162	3 (1.9)	0.4–5.3		0.1	0.0–0.4	0.001
	3: Makoni	64	1 (1.6)	0.0–8.4		0.1	0.0–0.8	0.026
	4: Abattoir	34	4 (11.8)	3.3–27.5		0.8	0.3–2.6	0.759
Species	1: Caprine	335	25 (7.5)	4.9–10.8	p = 0.282			
	2: Ovine	63	2 (3.2)	0.4–11.0				
Age	1: 1<x≤2 years	69	5 (7.3)	2.4–16.1	p = 0.913	Ref	-	-
	2: 2<x≤3 years	59	3 (5.1)	1.1–14.2		0.7	0.2–3.0	0.620
	3: 3<x≤4 years	93	5 (5.4)	1.8–12.1		0.7	0.2–2.6	0.630
	4: 4<x≤5 years	120	10 (8.3)	4.1–14.8		1.2	0.4–3.6	0.790
	5: x>5 years	57	4 (7.0)	2.0–17.0		1.0	0.3–3.8	0.960
Parity	0: 0	1	0	-	p = 0.339			
	1: 1–2	212	11 (5.2)	2.6–9.1				
	2: 3–4	144	12 (8.3)	4.4–14.1				
	3: ≥5	28	3 (10.7)	2.3–28.2				
Sex	1: Female	385	26 (6.8)	4.5–9.7	p = 0.605			
	2: Male	13	1 (7.7)	0.2–38.5				
Abortion history	0: No	281	21 (7.5)	4.7–11.2	p = 0.493			
	1: Yes	104	5 (4.8)	1.6–10.9				
Orchitis history	0: No	6	0	-	p = 1.000			
	1: Yes	7	1 (14.3)	0.4–57.9				

***N*,** number of animals; ***P***, probability value; ***CI***, Confidence Interval

* These variables had Fisher’s Exact *p* < 0.05 and were used in the univariable logistic regression analysis

^**d**^ Dependent variable: small ruminant seropositivity for *Toxoplasma gondii* (negative = 0, positive = 1); [%] test adjusted prevalence.

**Table 3:** Summary for descriptive and analytic statistics for *C*. *abortus* seropositivity in small ruminants from selected smallholder farming areas of Zimbabwe (2020).

Variable	Level	Distribution^d^			Association testing^d^
		*N*	*Prevalence (%)*	*CI (95%)*	*Fisher’s exact*
Seropositivity	Overall	398	8 (2.0) [0.6]	0.9–3.9	-
Location*	1: Chivi	138	7 (5.1)	2.1–10.2	p = 0.006
	2: Zvimba	162	0	-	
	3: Makoni	64	0	-	
	4: Abattoir	34	1 (2.9)	0.1–15.3	
Species	1: Caprine	335	8 (2.4)	1.0–4.7	p = 0.366
	2: Ovine	63	0	-	
Age	1: 1<x≤2 years	69	0	-	p = 0.703
	2: 2<x≤3 years	59	2 (3.4)	0.4–11.7	
	3: 3<x≤4 years	93	2 (2.2)	0.3–7.6	
	4: 4<x≤5 years	120	3 (2.5)	0.5–7.1	
	5: x>5 years	57	1 (1.8)	0.0–9.4	
Parity	0: 0	1	0	-	p = 0.851
	1: 1–2	212	4 (1.9)	0.5–4.8	
	2: 3–4	144	4 (2.8)	0.8–7.0	
	3: ≥5	28	0	-	
Sex	1: Female	385	8 (2.1)	0.9–4.1	p = 1.000
	2: Male	13	0	-	
Abortion history	0: No	281	8 (2.9)	1.2–5.5	p = 0.115
	1: Yes	104	0	-	

***N*,** number of animals; ***P***, probability value; ***CI***, Confidence Interval

* These variables had Fisher’s Exact *p* < 0.05 and were used in the univariable logistic regression analysis

^**d**^ Dependent variable: small ruminant seropositivity for *Chlamydia abortus* (negative = 0, positive = 1); [%] test adjusted prevalence.

### Questionnaire survey

**Fig 2:**
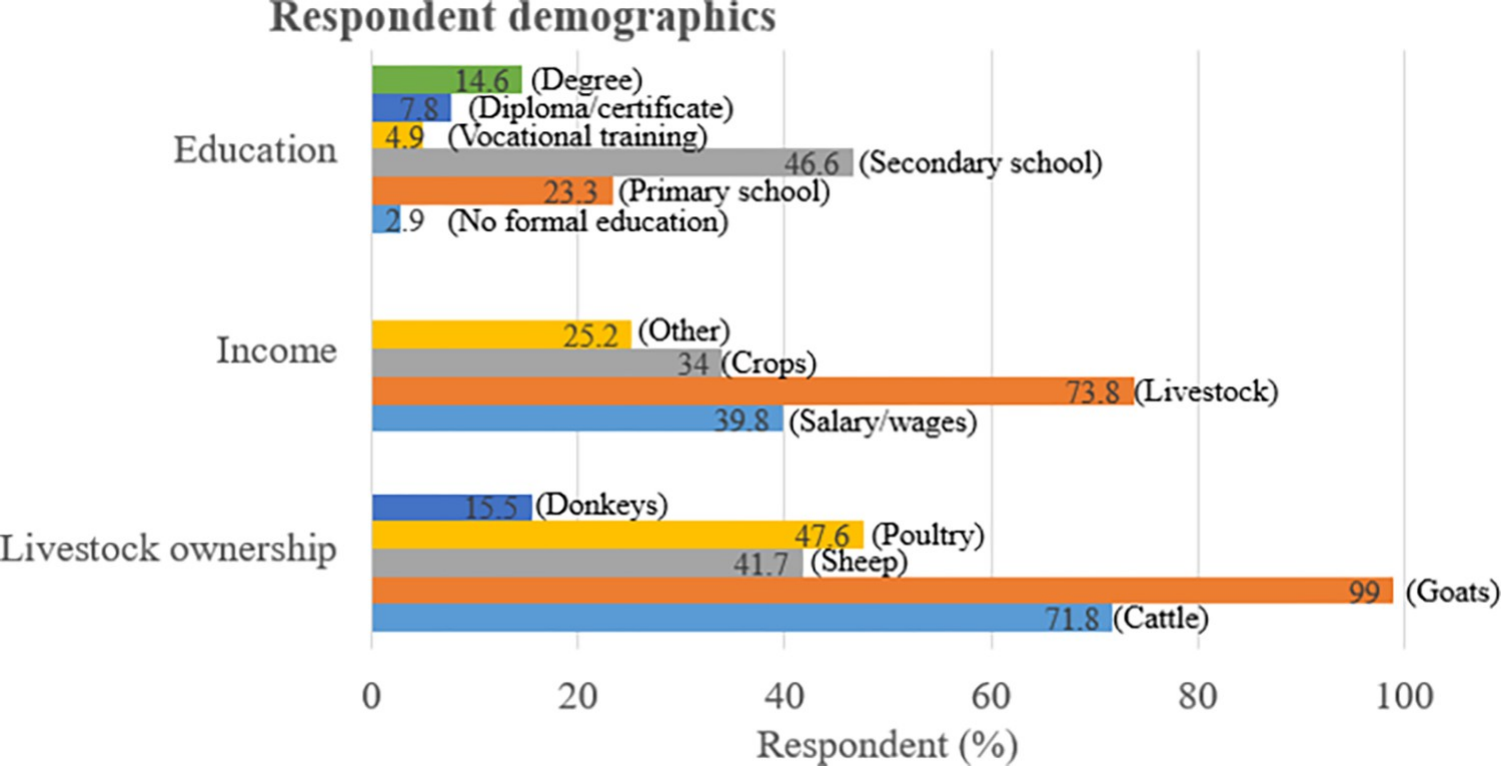
Demographics of interviewed smallholder livestock keepers by education, income, and livestock ownership.

**Fig 3:**
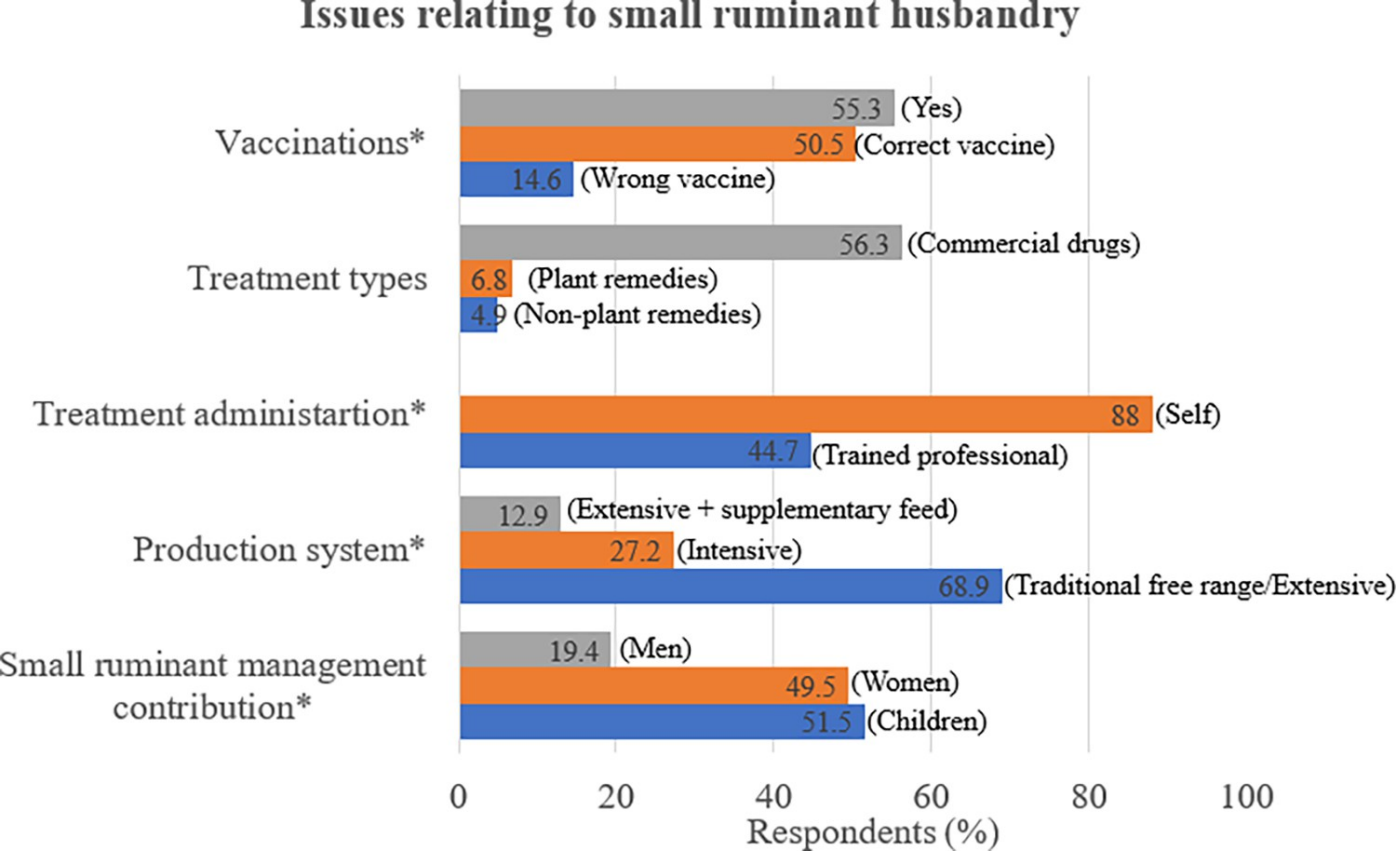
Issues relating to small ruminant husbandry. **NB:** All questions with * in this table where multiple choice (that is it was possible to select more than one ticked answer).

Males were the majority of interviewed respondents (72.8%, 75/103) compared to females (27.2%, 28/103). Most respondents (97.1%, 100/103) had received at least primary school education, but none indicated receiving any specialist training in agriculture. All respondents owned livestock, with goat ownership being the highest (99%) amongst the livestock species. Most respondents (73.8%) indicated that livestock production was an important source of cash income. The traditional free-range production system (68.9%) was predominant in all study areas. With children (51.5%) and women (49.5%) playing a major role in small ruminant husbandry. See Table 4, Figs 2 and 3.

**Table 4:** Demographics of interviewed smallholder livestock keepers by gender, household head, age, and household size.

Variable	Sex		Variable			
	Male	Female		Min	Mean	Max
Gender (%)	75 (72.8)	28 (27.2)	Age (years)	19	41.9	85
Household head (%)	102 (99)	1 (1)	Household size	3	5.9	16

**Fig 4:**
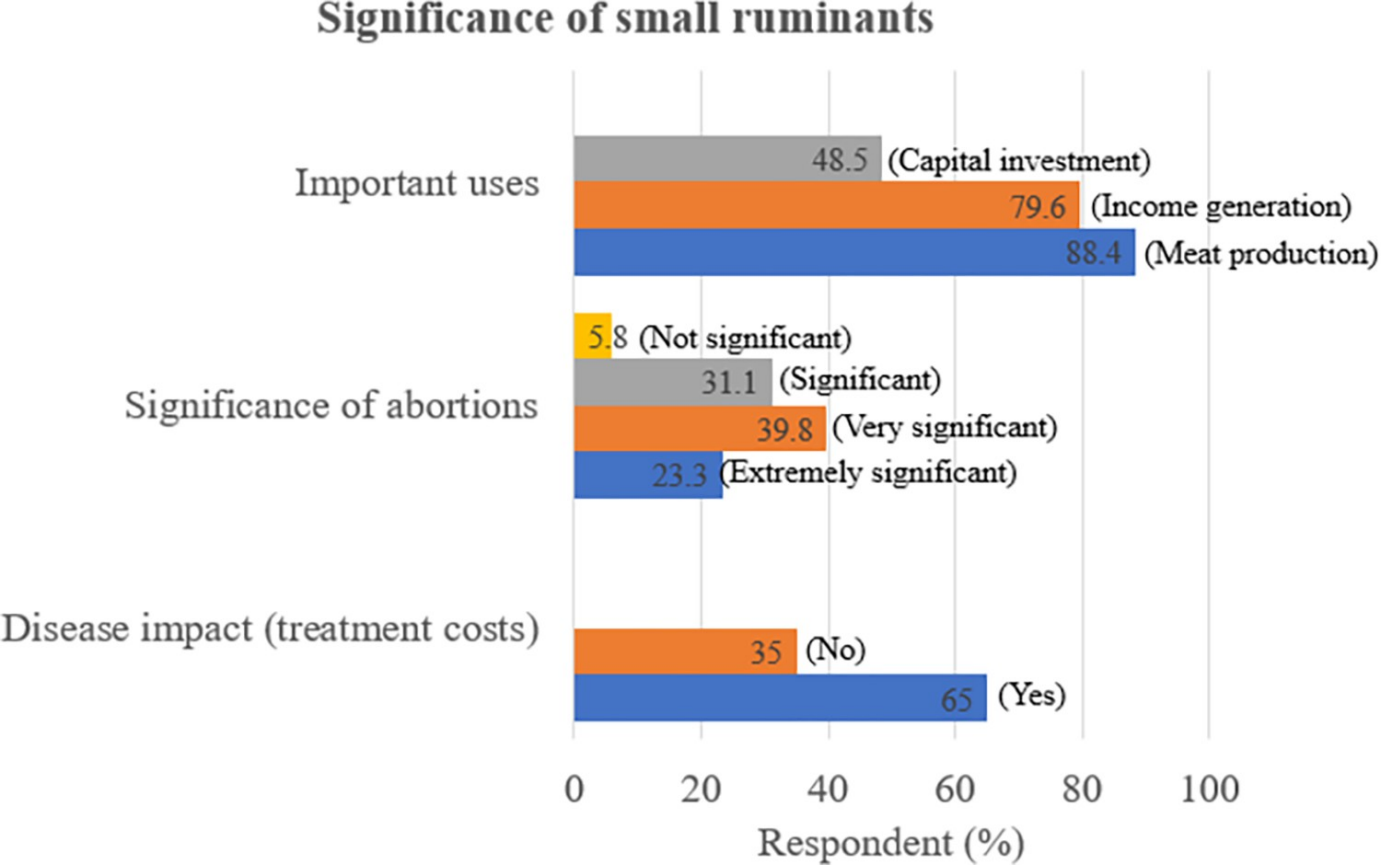
Summary of responses on the significance of small ruminants as indicated by farmer percentages. These where multiple-choice questions (that is it was possible to select more than one ticked answer).

43.7% of respondents reported having recently faced challenges with reproductive diseases within their flocks. Only 34% cited either an infectious or non-infectious cause of abortion in small ruminants. Generally, knowledge of *Brucella* spp. (9.7%), *C*. *abortus* (5.8%) and *T*. *gondii* (3.9%) in small ruminants was low. Overall, 14.6% believed humans were susceptible to these infections, but knowledge on *Brucella* spp. (5.8%), *C*. *abortus* (4.9%) and *T*. *gondii* (2.9%) symptoms in humans was low, see Table 5. 45.6% of respondents indicated that they consumed goat/sheep meat after roasting, while 18.5% consumed goat milk raw. 37.9% of respondents stated that they assisted in dystocia’s, and risky practices such as the use of bare hands when handling abortion contents and poor abortion contents disposal were stated by 40.8% and 10.7% (pet food (7.8%) and others (2.9%)) of respondents, respectively, see Table 6. The majority of respondents indicated that they mainly use small ruminants for meat (88.4%) and income generation (79.6%). Several respondents (65%) indicated that there was a significant cost associated with the treatment of small ruminant diseases including reproductive conditions, and 23.3% indicated that abortions were extremely significant to their small ruminant production. See Fig 4 for a summary of responses on importance of small ruminant reproductive failures on small ruminant production.

**Table 5:** Summary of responses relating to awareness of small ruminant abortion causes and zoonotic risk.

		Location			
		Chivi district (n = 37)	Makoni district (n = 31)	Zvimba district (n = 35)	Overall (n = 103)
Question	Responses	Farmers (%)	Farmers (%)	Farmers (%)	Farmers (%)
Recent reproductive disease challenge	Yes	22 (59.5)	9 (29)	14 (40)	45 (43.7)
Know abortion causes	Yes	11 (29.7)	15 (48.4)	9 (25.7)	35 (34)
Heard of *Brucella* spp. before	Yes	4 (10.8)	4 (12.9)	2 (5.7)	10 (9.7)
Heard of *C*. *abortus* before	Yes	3 (8.1)	3 (9.7)	0 -	6 (5.8)
Heard of *T*. *gondii* before	Yes	2 (5.4)	2 (6.5)	0 -	4 (3.9)
Have idea of *Brucella* spp. susceptible animals	Yes	14 (37.8)	14 (45.2)	9 (25.7)	37 (35.9)
Have idea of *C*. *abortus* susceptible animals	Yes	13 (35.1)	12 (38.7)	11 (31.4)	36 (35)
Have idea of *T*. *gondii* susceptible animals	Yes	12 (32.4)	11 (35.5)	7 (20)	30 (29.1)
Knowledge of how *Brucella* spp. infects animals	Yes	2 (5.4)	5 (16.1)	5 (14.3)	12 (11.7)
Knowledge of how *C*. *abortus* infects animals	Yes	1 (2.7)	4 (12.9)	3 (8.6)	8 (7.8)
Knowledge of how *T*. *gondii* infects animals	Yes	0 -	3 (9.7)	2 (5.7)	5 (4.9)
Are humans susceptible to *Brucella* spp., *C*. *abortus* & *T*. *gondii*	Yes	10 (27)	2 (6.5)	3 (8.6)	15 (14.6)
Know *Brucella* spp. symptoms in humans	Yes	2 (5.4)	2 (6.5)	2 (5.7)	6 (5.8)
Know *C*. *abortus* symptoms in humans	Yes	2 (5.4)	2 (6.5)	1 (2.9)	5 (4.9)
Know *T*. *gondii* symptoms in humans	Yes	1 (2.7)	2 (6.5)	0 -	3 (2.9)

**Table 6:** Summary of responses relating to risky practices for contracting zoonoses.

		Location			
		Chivi district (n = 37)	Makoni district (n = 31)	Zvimba district (n = 35)	Overall (n = 103)
Variable	Responses	Farmers (%)	Farmers (%)	Farmers (%)	Farmers (%)
Milk preparation method prior to consumption	Raw	9 (24.3)	1 (3.2)	9 (25.7)	19 (18.5)
	Boiling	12 (32.4)	6 (19.4)	8 (22.9)	26 (25.2)
	Fermenting	2 (5.4)	2 (6.5)	1 (2.9)	5 (4.9)
Meat preparation method prior to consumption	Boiling	37 (100)	25 (80.7)	29 (82.9)	91 (88.4)
	Drying	32 (86.5)	16 (51.6)	14 (40)	62 (60.2)
	Roasting	14 (37.8)	17 (54.8)	16 (45.7)	47 (45.6)
	Chilling	2 (5.4)	0 -	2 (5.7)	4 (3.9)
Assistance in dystocia	Yes	9 (24.3)	18 (58.1)	12 (34.3)	39 (37.9)
	Birth aid	8 (21.6)	18 (58.1)	11 (31.4)	37 (35.9)
	Assist vet	3 (8.1)	7 (22.6)	3 (8.6)	13 (12.6)
Protective methods used when handling abortion contents	Gloves	9 (24.3)	11 (35.5)	9 (25.7)	29 (28.2)
	Bare hands	20 (54.1)	12 (38.7)	10 (28.6)	42 (40.8)
	Wash hands	4 (10.8)	17 (54.8)	18 (51.4)	39 (37.9)
	Other*	4 (10.8)	4 (12.9)	4 (11.4)	12 (11.7)
Disposal methods of abortion contents	Bury	34 (91.9)	31 (100)	28 (80)	93 (90.3)
	Burn	14 (37.8)	22 (71)	25 (71.4)	61 (59.2)
	Pet food	0 -	6 (19.4)	2 (5.7)	8 (7.8)
	Other**	2 (5.4)	1 (3.2)	0 -	3 (2.9)

Other

*: Do nothing, use shovel, use plastics/paper/sacks, ***Other***

**: throw in pit, dispose far away from homestead, do nothing. **NB:** All questions in this table were multiple choice (that is it was possible to select more than one ticked answer).

## Discussion

The present study demonstrated the first serological evidence of brucellosis in small ruminants in Zimbabwe since 1996, after Zimbabwe eliminated the disease [1]. This study also builds the evidence on small ruminant toxoplasmosis and chlamydiosis in Zimbabwe, with the detection of a surprisingly low overall toxoplasmosis seroprevalence compared to previous studies [5]. The questionnaire survey revealed that respondents had little knowledge of brucellosis, chlamydiosis, and toxoplasmosis in small ruminants, and that most respondents were at risk of exposure to zoonoses through risky animal husbandry practices and poor handling of animal products.

Appropriate assay selection informed by a literature review is important, as diagnostic assays utilised in most surveys have diagnostic sensitivities and specificities of less than 100% [24]. This may result in false positive or false negative misclassification(s) [24, 28]. As such, in this study to account for “imperfect” test bias we adjusted the seroprevalences to factor-in the assay sensitivities and specificities. However, there were no notable differences between the seroprevalence estimates and the test-adjusted seroprevalences for both brucellosis and toxoplasmosis. While the chlamydiosis seroprevalence declined from 2.0% to a test-adjusted estimate of 0.6%. This may be due to the use of an iELISA which had a moderate sensitivity. This test was selected because it was better than the Complement Fixation Test and crude ELISAs which are no longer recommended because they lack specificity and thus cannot differentiate cross-reacting antibodies between *C*. *abortus* and other *Chlamydia* species or some Gram-negative bacteria [29]. However, this iELISA had a high specificity, and utilised a synthetic MOMP antigen which is more specific for *C*. *abortus* antibodies thus minimising cross reactions between *C*. *abortus* and other *Chlamydia* species or some Gram-negative bacteria [29, 30].

For brucellosis seroprevalence determination, a highly sensitive and specific iELISA assay was used which minimised false positive reactions from cross-reacting Gram-negative bacteria [31]. Such iELISA assays have been shown to be reliable stand-alone tests with better performance when compared to classical serial testing [31–34], furthermore they are prescribed tests for international trade by the World Organisation for Animal Health [35, 36]. While iELISA does not differentiate antibody cross-reactions between *B*. *abortus*/*melitensis* [32, 37], *B*. *abortus* has not yet been isolated from small ruminants in Zimbabwe [38–40]. For toxoplasmosis seroprevalence determination a highly sensitive and specific iELISA assay, which is a recognised test method was used [41]. Currently vaccination against *Brucella* spp., *T*. *gondii*, and *C*. *abortus* in small ruminants are not practiced in Zimbabwe [19, 38]. Therefore, the seropositive results obtained in the present study are believed to be from natural infection(s). However, the most likely *Brucella* spp. detected here is *B*. *melitensis* [26, 39, 42].

The 9.1% *Brucella* spp. seroprevalence recorded in this study is comparable to other small ruminant studies elsewhere in Africa [11, 32, 43–46]; conversely, studies in 2013 [47] and 2019 [19] did not detect evidence of brucellosis in small ruminants in other parts of Zimbabwe. This difference could be due to differences in the methodologies, husbandry practices, and the possibility of absence and/or low levels of disease in the various geographical locations sampled, in each study respectively [1, 8, 19]. In this study seroreactors were not present in all study areas, although location showed a statistical association with seropositivity. This geographical heterogeneity could be explained by differences in husbandry practices and other agroecological factors that may influence brucellosis disease dynamics [1, 8, 21]. Additionally, this finding could suggest that the sampled populations in those locations which did not record any seroreactors may be naturally free from brucellosis or that brucellosis prevalence(s) are very low. The difference in seroprevalence observed between the abattoir (Goromonzi district) (76.5%, OR = 25.6, 95% CI 7.4–89.2) and Makoni district (15.6%) could be due in part to 1) differences in husbandry practices, as most of the small ruminants from abattoirs were probably from commercial farms, where semi-intensive/intensive husbandry is predominant [20, 21, 48], and 2) the abattoir sample was comprised of female animals which are usually culled either due to old age, disease(s) or poor reproductive performance. If this assumption is true, this could have substantially increased the odds of picking an infected animal from the abattoir. A similarly high seroprevalence has also been recorded at an abattoir related study in Namibia (61.9% (26/42 goats)) [32].

The steady increase in both *Brucella*-seropositivity and odds of infection observed with age (OR = 7.3, 95% CI 2.5–21.6) and parity (OR = 2.9, 95% CI 1.4–6.0) in the univariable analysis concurred with other studies [20, 21, 32, 49]. This increase might be due to increased cumulative exposure as age increases, also the onset of sexual maturity has been linked with an increased risk/susceptibility to *Brucella* spp. infection especially following abortion storms [8, 20, 21, 48, 49]. Animals with abortion history showed higher odds (OR = 3.1 (95% CI 1.5–6.1), univariable logistic regression analysis) of *Brucella*-seropositivity. This concurs with findings from other ruminant studies [7, 8, 20, 21, 48, 49]. In 1987 Halliwell *et al*., [50] identified *B*. *melitensis* (later identified as *B*. *melitensis* biovar 1 [40]) in goats that were believed to have been illegally translocated from Mozambique into Zimbabwe [39]. The detection of caprine brucellosis illustrated in this study could be due to similar circumstances of transhumance and/or commercial movements/importations of unknown *Brucella*-status small ruminants from neighbouring countries for the purposes of restocking and improving flock genetic material [48, 51–54]. That, coupled with the lack of organized caprine/ovine brucellosis surveillance and control measures in Zimbabwe, could have resulted in the reintroduction of this disease [38, 55]. This is a further reminder for the need of regular serological surveillance for caprine/ovine brucellosis and the enforcement of stringent livestock importation and movement regulations in Zimbabwe. Unfortunately, investigating the source/origin of caprine brucellosis was beyond the scope of this study.

The 6.8% toxoplasmosis seroprevalence recorded in this study is comparable to findings reported by Penzhon & Van Knapen [56] (9.2% (sheep) and 2.9% (goats)) from different parts of Zimbabwe. Conversely, Hove *et al*., [5] reported a much higher overall seroprevalence of 67.2% in small ruminants also from Zimbabwe. This difference could be due to differences in; husbandry practises, geographical locations and climates, cat population densities, age and sex of sampled animals and the methodologies employed, in each respective study [14, 57–62]. Changes in disease dynamics over time could also account for such differences [63], as there are 15/16-years between the current study and the 2005 [5] study. In the 15/16-years Zimbabwe has experienced multiple drought events (most recent occurred in 2018–2019) [64] that could significantly influence changes in *T*. *gondii* disease dynamics, as prolonged spells of arid conditions do not favour *T*. *gondii* oocyst sporulation and long-term environmental survival [5, 10, 63, 65]. Furthermore, most of the animals sampled in the present study were extensively reared which could also account for such a low overall seroprevalence [5, 10, 18, 63, 65, 66]. Similarly low seroprevalences have been reported in other southern African countries; 10% (goats) Botswana [67]; 6.4% (sheep) South Africa [18]; 4.3% (goats) South Africa [68]; 9.2% (goats) South Africa [6]; and 8% (sheep) South Africa [66], however, reports of high seroprevalences also exist; 30% (goats) Botswana [14], and 64.5% (sheep) and 53.9% (goats) South Africa [60]. These contrasting findings indicate a wide distribution of small ruminant toxoplasmosis, pointing to the influence of a variety of factors in the disease epidemiology both in Zimbabwe and southern Africa.

In the present study, location showed a significant association with *T*. *gondii* seropositivity, which concurs with other studies [58, 60, 69]. *T*. *gondii* appears to be present in all study locations but at varying levels. This seroprevalence trend is similar to that reported by Hove *et al*., [5], with animals sampled from the rural area (Chivi district) recording the highest prevalence, this is also in agreement with other southern African studies [18, 60]. This trend could be due to increased livestock, crop, and human pressure on limited land in rural areas, which results in animals grazing around households where cat densities, and concentration of domestic cat defecation sites and faeces are probably highest [5, 58, 70, 71]. Zvimba and Makoni districts are both located in the cooler sub-humid to humid areas of Zimbabwe [23] with conditions conducive for *T*. *gondii* oocyst sporulation and survival [5, 65, 72], however, both districts recorded low seroprevalences, which is contrary to findings by other authors of studies in similar climatic conditions [5, 18, 72]. This could be associated with other factors such as extensive rearing plus larger landholding in resettlement areas of both districts (up to 35 Hectares) [15, 18, 22, 66], where animals graze relatively larger pastures and domestic cats are usually restricted to and around households while wild cats are rare/absent, thus environmental *T*. *gondii* oocysts contamination may be low and/or highly dispersed [5, 10, 15, 58].

The seroprevalence of *C*. *abortus* (2.0%) in the present study appears to be low, this is contrary to previous findings by Bhandi *et al*., [19] (22%) in Zimbabwe. These differences could be due to differences in target populations, husbandry practices, climatic conditions and methodologies used in each study respectively [73–75]. There is no direct explanation for the low seroprevalence and the source/origin of the *C*. *abortus* infection observed in the present study. However, the presence of chlamydial infection could be associated with the possibility of an independent chlamydial infection cycle circulating among domestic ruminants (that does not involve wild ruminant species) as previously suggested by other authors in Zimbabwe [12, 19]. Also, because introduction of chlamydial infections into flocks and locations is dependent on transhumance activities [25, 76–79], husbandry practices could have influenced these findings as some of the sampled animals could have been from closed flocks and/or from areas where the disease is absent or occurs at very low levels. A statistical association was observed between seropositivity and location, similar findings were also observed by Al-Qudah *et al*. [78] in Jordan and Tesfaye *et al*. [73] in Tunisia. However, not all locations had seroreactors. This geographical heterogeneity could be due to variations in environmental conditions and husbandry practices such as the frequency of introduction of new animals with unknown *C*. *abortus*-status into flocks (closed vs open flocks), as well as the frequency of inter-flock mixing with neighbouring flocks [25, 76–80]. The lack of seroreactors in Zvimba and Makoni districts is suggestive that the sampled populations might have been naturally free from the disease or that very low chlamydial infection pressures exist.

Although both *C*. *abortus* and *T*. *gondii* infections are known to cause small ruminant abortions [19, 25, 81] there was no statistical association observed with abortion history in the present study. Similar findings for *C*. *abortus* were also observed in other studies in Zimbabwe by Bhandi *et al*., [19] in goats and Ndengu *et al*., [12] in cattle and elsewhere for *T*. *gondii* [10, 58]. These finding suggest that *C*. *abortus* and *T*. *gondii* play a minor role in small ruminant abortions in the sampled populations in this study and could imply the presence of other abortion causing agents [10, 58] that may require investigation.

The questionnaire survey established that most respondents had received at least primary school education, which is suggestive of a relatively reasonable level of understanding of the questionnaire. The survey established that most households depend on livestock production as an important source of food security and income, with goat production playing a significant role in their livelihoods as evidenced by the high goat ownership observed. Similar findings were also observed by Homann *et al*. [2]; Ndengu *et al*. [17]; and Mhlanga *et al*. [3] in other parts of Zimbabwe. The main findings of this survey are that small ruminant abortions and reproductive failures are experienced by farmers in all study areas and that just under 45% of farmers indicated having recently faced challenges with reproductive diseases in their flocks. This agrees with other studies that have been carried out in cattle in Zimbabwe [17]. Despite this experience, knowledge of abortion causing agents in small ruminants was low. This could be due to lack of adequate investigations into abortions by veterinary authorities due to inadequate diagnostic resources. Additionally, in the rare case of an investigation, the results are often inconclusive and/or rarely/poorly relayed to farmers [4, 17]. This study also exposed that knowledge on the zoonotic potential, including the transmission pathways and symptoms in both livestock and humans of *Brucella* spp., *C*. *abortus* and *T*. *gondii* was very low, this is consistent with other African studies [6, 16, 17, 20, 82, 83]. This could be due to poor access to veterinary services by smallholder farmers and hence there is a clear need to promote education on the associated risk factors of zoonotic disease among at risk communities, through a coordinated One Health approach. The survey revealed that most farmers rely on communal pastures for their small ruminant production (traditional free-range system), similar findings were also reported by ZimStat & World Bank [84] and Ndlovu *et al*. [4] in other parts of Zimbabwe. This sharing of pastures between flocks/herds from different household has been shown to be a significant risk factor in the spread of diseases like brucellosis or chlamydiosis [25–27, 75, 80]. As reported elsewhere children and women were also shown to play a major role in small ruminant husbandry in the present study, putting them at risk of encountering zoonotic infections [2–4]. The practice of consuming roasted meat cited by several respondents, indicates a possible risk of infection with *T*. *gondii* and infrequently *Brucella* spp. (higher bacterial concentrations in kidney, udder, testis, and liver [33]) as roasting might not render the meat free from bacteria or parasite depending on how “rare” the meat is [6, 85, 86]. Although goat milk is rarely consumed, a few farmers indicated that they consume it raw, previous studies have shown that goat milk consumption is mainly reserved for the elderly and infants [2, 3, 5], this puts them at significant risk of *T*. *gondii* and/or *Brucella* spp. infection [5, 32, 33, 85, 86]. Also, several farmers indicated that they took part in assisting in dystocia’s as well as handled abortion contents with little or no protection. This has been shown in previous studies to significantly increase the risk of human brucellosis or chlamydiosis [20, 75, 87]. Education on good hygienic practices, food safety measures, and wearing personal protective equipment while gardening, handling animals and/or animal waste/secretions is indicated to reduce the risk of infection [6, 33]. Regular serological screening of food animals and the contact human population is vital in determining the public health consequences of these infections [6]. Most farmers indicated that they use small ruminants primarily for meat and income generation among other uses, similar findings were also reported in other studies in Zimbabwe [2–4]. Most farmers indicated that there was a significant cost associated with the treatment of small ruminant diseases including reproductive conditions, and that abortions were significantly affecting their small ruminant production. Similar findings on neonatal mortalities have been reported in other studies in Zimbabwe [4]. Although this study did not investigate the expenses covered by income generated from small ruminant sells, the high goat ownership plus the main uses cited by farmers (meat and income) is indicative of the importance of small ruminant production to their livelihoods, similar findings were also observed by Homann *et al*. [2]. Thus, further studies on the socioeconomic impact of reproductive losses and the economic burden of disease induced by zoonotic abortion causing agents are indicated.

### Limitations of the study

There are some further limitations to this study. Abattoir data may not be truly representative of the population [88], as it may introduce bias through the sampling of less healthy or poor producing, or old animals sent for slaughter. Furthermore, only female animals were sampled from the abattoir in the present study which might have also introduced bias to the abattoir sample (females are usually culled for either poor reproductive performance or old age). The knowledge of animal reproductive status was inaccurate as farmers mainly relied on recall due to absence of written production records for their flocks. Hence it was not possible to accurately link with confidence a causal relationship between the stated abortion history and the obtained seroprevalence(s). Because of the limited time scale and resources, it was not possible to formally pre-test the questionnaire this could have significantly influenced the quality of responses that were received.

## Conclusion

The serological evidence of caprine brucellosis illustrated here, shows the need to extend the existing brucellosis control program, which only caters for cattle, to include sheep and goats for efficient surveillance and control. Further studies are required to determine the *Brucella* spp. detected here to its exact species level, since brucellosis control measures are dependent on the infecting species. More studies are required to determine the exact role these three agents play in small ruminant reproductive losses, as well as to determine their economic burden of disease both in humans and livestock. Lastly the paucity of knowledge of zoonotic causes of abortion in small ruminants demonstrated here warrants the need to teach smallholder farmers of the risk factors of these diseases in production animals so that risks can be understood and mitigated.

## Supporting information

S1 Table 2019 Livestock census by province, Zimbabwe. (DOCX)

S2 Table Study sites goat and sheep total populations together with provincial totals and their respective agroecological regions. Livestock census, Central statistics office DLVS.
(DOCX)

S1 File Questionnaire to assess the knowledge, attitude, and practices of farmers towards small ruminant abortions in selected smallholder commercial farming areas of Zimbabwe. (DOC)

S2 File Questionnaire dataset: Questionnaire raw data used for analysis. (XLSX)

S3 File Questionnaire data dictionary: Used to code responses. (XLSX)

S4 File Serology dataset: Serology raw data used for analysis. (XLSX)

S5 File Toxop lasmosis iELISA package insert: ID Screen® Toxoplasmosis Indirect Multi-species. (PDF)

S6 File Chlamydiosis iELISA package insert: ID Screen® *Chlamydophila abortus* Indirect Multi-species. (PDF)

S7 File Brucellosis iELISA package insert: The ID Screen® brucellosis Serum Indirect Multi-species. (PDF)

S8 File Inclusivity in global research statement: Questionnaire-typeset39MIO. (DOCX)
