# Supplementary material for: Seroprevalence and assessment of public awareness of Brucella spp., Toxoplasma gondii and Chlamydia abortus in small ruminants from selected smallholder commercial farms of Zimbabwe
Source: PLoS One. 2023 Jun 29;18(6):e0287902. doi: 10.1371/journal.pone.0287902 (PMC10310052; doi:10.1371/journal.pone.0287902)
Supplement: S1 Table — (DOCX) [file pone.0287902.s001.docx]

**Supplemental table**

| **S1:** 2019 Livestock census by province, Zimbabwe. | | | | | |
| --- | --- | --- | --- | --- | --- |
| **Province** | **Cattle** | **Sheep** | **Goats** | **Pigs** | **Donkeys** |
| Manicaland**^2^** | 606,882 | 100,462 | 634,742 | 41,630 | 39,200 |
| Mashonaland central | 562,630 | 60,123 | 264,616 | 58,821 | 6,438 |
| Mashonaland east**^4^** | 600,120 | 24,844 | 166,532 | 31,259 | 9,160 |
| Mashonaland west**^3^** | 639,038 | 51,244 | 185,593 | 151,279 | 15,903 |
| Masvingo**^1^** | 1,005,118 | 83,829 | 574,814 | 69,298 | 73,898 |
| Matabeleland north | 448,710 | 37,885 | 236,897 | 11,282 | 45,737 |
| Matabeleland south | 1,009,106 | 1,480,044 | 417,920 | 28,498 | 114,975 |
| Midlands | 707,964 | 16,341 | 289,180 | 20,994 | 61,974 |
| **Grand Total** | **5,579,568** | **1,854,772** | **2,770,294** | **413,061** | **367,285** |
| **^1,2,3,4^:** indicates which provinces the sampled districts came from (**^1^**Chivi, **^2^**Makoni, **^3^**Zvimba, and **^4^**Goromonzi. | | | | | |
