# Supplementary material for: Seroprevalence and assessment of public awareness of Brucella spp., Toxoplasma gondii and Chlamydia abortus in small ruminants from selected smallholder commercial farms of Zimbabwe
Source: PLoS One. 2023 Jun 29;18(6):e0287902. doi: 10.1371/journal.pone.0287902 (PMC10310052; doi:10.1371/journal.pone.0287902)
Supplement: S2 Table — Livestock census, Central statistics office DLVS. (DOCX) [file pone.0287902.s002.docx]

**Supplemental table**

| **S2:** Study sites goat and sheep total populations together with provincial totals and their respective agroecological regions. Livestock census, Central statistics office DLVS. | | |
| --- | --- | --- |
| **Study site** | **Province (2019)** | **Agroecological region** |
| Rural area  Chivi district (2020) | Masvingo* | Region IV and V |
| Goats: 100,095  Sheep: 6,646 | Goats: 574,814  Sheep: 83,829 |  |
| Resettlement small-scale  Makoni district (2020) | Manicaland* | Region IIb |
| Goats: 87,624  Sheep: 4,223 | Goats: 634,742  Sheep: 100,462 |  |
| Resettlement small-scale  Zvimba district (2020) | Mashonaland West** | Region IIa |
| Goats: 40,000  Sheep: 12,500 | Goats: 185,593  Sheep: 51,244 |  |
| Grade B Abattoir  Goromonzi district (2019) | Mashonaland East** | Region IIa |
| Goats: 15,670  Sheep: 2,241 | Goats: 166,532  Sheep: 24,844 |  |
| *These provinces are in the top 3 provinces with the largest small ruminant population densities nationally. ** These provinces have the lowest small ruminant population densities nationally. | | |
