# Supplementary material for: Seroprevalence and assessment of public awareness of Brucella spp., Toxoplasma gondii and Chlamydia abortus in small ruminants from selected smallholder commercial farms of Zimbabwe
Source: PLoS One. 2023 Jun 29;18(6):e0287902. doi: 10.1371/journal.pone.0287902 (PMC10310052; doi:10.1371/journal.pone.0287902)
Supplement: S1 File — (DOC) [file pone.0287902.s003.doc]

**Questionnaire to assess the knowledge, attitude, and practices of farmers towards small ruminant abortions in selected smallholder commercial farming areas of Zimbabwe.**

Dear participant,

This questionnaire will be used to collect information on the knowledge, attitudes, and practices used by small ruminant farmers in the small-holder sector of small ruminant production on small ruminant abortions in Zimbabwe as well as to assess the impact of small ruminant abortions on these communities. Results of this study will help inform the design of control programmes for animal and human chlamydiosis brucellosis and toxoplasmosis.

Information generated through this questionnaire will be used only for the purposes of this research, there are no right or wrong answers. Participation in this study is voluntary. There is no need to tell your name to the enumerator. Your responses will be kept confidential. You will in no way be personally linked to any of the results of the survey. There is no risk to you from participating in this questionnaire, and there is no anticipated direct benefit. Thank you in advance for your participation!

**SECTION A: DEMOGRAPHIC ISSUES**

**1.** Location:

**2.** Sex of respondent: Male Female

**3.** Age of respondent:

**4.** Level of education: **No formal education Primary school**  **Secondary/High school**

**Technical /vocational training**  **Diploma/Certificate University degree Don’t know Refused**

**5.** Who is the head of the household? (1) Father (2) Mother (3) Children

(4) Other (specify)

**6.** How many people live in your household (children and relatives included)?

**7.** Sources of income? (1) Crops (2) livestock (3) salaries/wages

(4) Other (specify)

**8.** How many animals do you have altogether?

Cattle Poultry Pigs Sheep Goats

Other (specify)

**SECTION B: KNOWLEDGE/AWARENESS**

1. What are the common diseases that affect your small ruminants?

………………………………………………………………………………………………………………………

1. Do you have problems with reproductive diseases in your flock? Yes No
2. Of the reproductive conditions that affect your small ruminants fill in the following table and rank them according to the importance from **1** (very important) to **5** (least important)

| **Condition/Disease** | **Frequency of occurrence** | **Rank** |
| --- | --- | --- |
| Abortion |  |  |
| Still born |  |  |
| Birth of weak offspring |  |  |
| Swollen testis |  |  |
| Other (specify)  ……………………… |  |  |

1. What do you associate small ruminant abortions (abortion storms) with?

………………………………………………………………………………………………………………….

1. Have you heard of the following disease conditions: Brucellosis, Chlamydiosis, and Toxoplasmosis?

Only 1 Only 2 All None

If yes, fill in the table below

| **Disease condition** | Where did you get the information?  (1) Veterinary doctors (2)Extentionist  (3) Newspapers/magazine (4)Television/radio  (5) Internet (6)From this study  (7) Other (Specify) |
| --- | --- |
| Brucellosis |  |
| Chlamydiosis |  |
| Toxoplasmosis |  |

1. Which animals can be infected by:

Brucellosis…………………………………………………………………………………………………

Chlamydiosis………………………………………………………………………………………………

Toxoplasmosis…………………………………………………………...……………………………….

1. Do you know how animals are infected by the following? Yes No

(If yes, please explain how?):

Brucellosis…………………………………………………………………………………………………

Chlamydiosis………………………………………………………………………………………………

Toxoplasmosis…………………………………………………………………………………………….

1. Can humans be infected with these diseases? Yes No Don’t know

If yes, what are the **symptoms** and **how do they get it**?

Brucellosis…………………………………………………………………………………………………

Chlamydiosis………………………………………………………………………………………………

Toxoplasmosis…………………………………………………………………………………………….

**SECTION** **C: ATTITUDES**

*Skip question 1, 2 and 3 if the answer was* ***NO*** *on the question “have you heard of the disease conditions: Brucellosis, Chlamydiosis, and Toxoplasmosis?” (Section B5)*

**1.** Do you believe any family members are at risk of acquiring brucellosis, chlamydiosis, or toxoplasmosis?

Yes No

If yes, which condition(s):…………………………………………………………………………………………..

**2.** If yes, to the above questions, which family member(s) do you think is/are most susceptible to infection?....................................................................................................................................................................

**3.** If any animal in your household gets brucellosis, chlamydiosis, or toxoplasmosis how serious do you consider this to be?

Fill in the table below with: Not serious, quite serious, very serious, or not applicable

| **Animal** | **Brucellosis** | **Chlamydiosis** | **Toxoplasmosis** |
| --- | --- | --- | --- |
| Goat |  |  |  |
| Sheep |  |  |  |

**4.** How significant are small ruminant abortions to your livelihood?

1. Extremely significant 2. Very significant 3. Significant

4. Not significant

**5.** Do you need/would you like more information on these diseases?

Yes No

If yes, how would you like to receive that information?............................................................................................

**SECTION** **D: PRACTICES**

**1.** Who is responsible for the day-to-day management of the small ruminants?

(1) Father (2) Mother (3) Grandfather (4) Grandmother (5) Child

(6) Other (specify)

**2.** What system do you use to manage the small ruminants? (1) Free range (2) intensive based on supplementation (3) mixed (4) Other (specify)

**3.** What supplementary feed do you give to your small ruminants? (1) None, (2) Stover (3) silage,

(4) Other (specify)

**4.** Please tick the function(s) and sub function(s) small ruminants play in your household in the table below.

| **Role/function** | **Sub function(s)** | | |
| --- | --- | --- | --- |
| Consumption | Milk | Meat | Use or sale of hides and/or horns |
|  |  |  |
| Household finance | Income generation | Bill payment (school fees, groceries, emergencies, etc) | Capital storage (investment) |
|  |  |  |
| Social | Installation of ancestral spirits | Cultural ceremonies | Social status (pleasure in ownership) |
|  |  |  |
| Relating to crop production | Provision of manure | | |
|  | | |
| Other (specify) |  |  |  |

**5.** When used for consumption, how do you process the animal products prior to consumption?

(State the animal product used for consumption in the corresponding box)

| **Method of processing** | Boiling | Drying | Roasting | Other method (specify)  ……………….………….. |
| --- | --- | --- | --- | --- |
| **Animal product(s)** |  |  |  |  |

**6.** Do you vaccinate your sheep/goats? 1. Yes 2. No

If yes, List the vaccinations and how often you do them?

…………………………………………………………………………………………………...……………………………………………………………………………………………………………………………………...….

**7.** Do you treat any diseases including reproductive conditions affecting your small ruminants?

(1) Yes (2) No

If yes, what do you use? Plants Commercial drugs Non-plant remedies

What is the estimated cost associated with the treatment(s)?....................................................................................

**8.** Who performs the treatment; use the key below.

Father Mother Extensionist Vet Other (specify) …………………………………

**9.** Have you ever used alternative methods for controlling reproductive diseases?

Yes No

If yes, state what you did?

……………………………………………………………………………………………………….……………...………………………………………………………………………………………………………………………

**10.** Do you assist delivery of pregnant sheep/goats? Yes No

If yes, how do you assist?

Birth aid Assistance to veterinarians Giving Intrauterine medication

**11.** Do you take any specific actions to protect yourself when dealing with sheep/goats having an abortion or with retained placenta/dead fetuses? **Don’t read out**.

Use gloves Use mask Wash hands Other (specify)

**12.** State how you dispose or handle the contents of the abortion after an abortion has occurred? **Don’t read out.**

Burn Bury Eat Pet food Other (specify)

**13.** Have you or a family member suffered from any diseases during the time of abortion storms or from handling small ruminants?

Yes No

If yes, state the following

Disease condition diagnosed…………………………………………………………………...

How condition was managed…………………………………………………………………...

How much treatment cost……………………………………………………………………….

| **THANK YOU** |  |
| --- | --- |
