# Supplementary material for: Seroprevalence and assessment of public awareness of Brucella spp., Toxoplasma gondii and Chlamydia abortus in small ruminants from selected smallholder commercial farms of Zimbabwe
Source: PLoS One. 2023 Jun 29;18(6):e0287902. doi: 10.1371/journal.pone.0287902 (PMC10310052; doi:10.1371/journal.pone.0287902)
Supplement: S6 File — (PDF) [file pone.0287902.s008.pdf]

General Information

Chlamydophila abortus is a major cause of abortion. Serological diagnosis may be achieved by the complement fixation test (CFT) or by ELISA. When these tests use LPS or whole bacteria as antigens, they generally present low specificity and sensitivity levels, and cross reactions are often observed with *Chlamydophila pecorum.*

The ID Screen® Chlamydophila abortus Indirect ELISA uses a synthetic antigen from a major outer-membrane protein (Momp) specific to *Chlamydophila abortus*, which reduces the frequency of non-specific reactions.

This kit can be used for a variety of species, including ruminants, horses and swine. Please contact IDvet for use in other species.

Description and Principle

Microwells are coated with a *Chlamydophila abortus*-specific antigen (Momp).

Samples to be tested and controls are added to the wells. Anti-*Chlamydophila* antibodies, if present, form an antigen-antibody complex.

After washing, a multi-species peroxidase (HRP) conjugate anti is added to the wells. It fixes to the anti-*Chlamydophila* antibodies, forming an antigen-antibody-conjugate-HRP complex.

After elimination of the excess conjugate by washing, the substrate solution (TMB) is added.

The resulting coloration depends on the quantity of specific antibodies present in the specimen to be tested. In the presence of antibodies, a blue solution appears which becomes yellow after addition of the stop solution. In the absence of antibodies, no coloration appears.

The microplate is read at 450nm.

Kit Components

**Reagents***
Microplates coated with a *Chlamydophila* antigen
Concentrated Conjugate (10X)
Positive Control
Negative Control
Dilution Buffer 13
Dilution Buffer 3
Concentrated Wash Solution (20X)
Substrate Solution
Stop Solution (0,5 M)

** Quantities supplied are indicated on the kit label.*

1. The conjugate, the controls and the substrate solution must be stored at 5°C (± 3°C).
2. The other reagents can be stored between +2°C and +26°C.
3. Components bearing the same name (wash solution, dilution buffers) can be used for the entire IDvet product range.

Materials required but not provided

1. Mono or multi-channel micropipettors capable of delivering volumes of 10 µl, 100 µl, and 200 µl.
2. Disposable tips.
3. 96-well microplate reader.
4. Distilled or deionized water.
5. Manual or automatic wash system.

Precautions

1. Do not pipette by mouth.
2. The substrate solution can be irritating to the skin.
3. The stop solution (0.5 M) may be harmful if swallowed. It may cause sensitisation by skin contact (**R22-43**). Avoid contact with skin (**S24-37**).
4. Do not expose the substrate solution to bright light nor to oxidating agents.
5. All single-use material used for the assays should be decontaminated by immersion in freshly prepared 5% sodium hypochlorite for minimum 1 hour before elimination, or by autoclaving at 120°C.

Sample Preparation

In order to avoid differences in incubation times between specimens, it is possible to prepare a 96-well plate containing the test and control specimens, before transferring them into an ELISA microplate using a multichannel pipette.

Wash Solution Preparation

If necessary, bring the Wash Concentrate **(20X)** to room temperature and mix thoroughly to ensure that the Wash Concentrate **(20X)** is completely solubilized.

Prepare the Wash Solution **(1X)** by diluting the Wash Concentrate **(20X)** to 1:20ème in distilled/deionized water.

Testing Procedure

Allow all the reagents to come to room temperature (21°C ± 5°C) before use. Homogenize all reagents by inversion or Vortex.

For serum:

1. Add 90 µl of **Dilution Buffer 13** to each microwell.
2. Add:
   - 10 µl of the **Negative Control** to wells A1 and B1.
   - 10 µl of the **Positive Control** to wells C1 and D1
   - 10 µl of each sample to be tested in the remaining wells.
3. Incubate **45 min** ± **4 min** at 21°C (± 5°C).
4. Empty the wells. Wash each well 3 times with approximately 300 µl of the **Wash Solution.** Avoid drying of the wells between washings.
5. Prepare the **Conjugate 1X** by diluting the **Concentrated Conjugate 10X** to **1:10** in **Dilution Buffer 3.**
6. Add 100 µl of the **Conjugate 1X** to each well.
7. Incubate **30 min** ± **3 min** at 21°C (± 5°C).
8. Empty the wells. Wash each well 3 times with approximately 300 µl of the **Wash Solution.** Avoid drying of the wells between washings.
9. Add 100 µl of the **Substrate Solution** to each well.
10. Incubate **15 min** ± **2 min** at 21°C (± 5°C) in the dark
11. Add 100 µl of the **Stop Solution** to each well in order to stop the reaction.
12. Read and record the O.D. at 450 nm.

Validation

The test is validated if:

✓ the mean value of the Positive Control O.D. (OD_{PC}) is greater than 0.350.

$$OD_{PC} > 0.350$$

✓ the ratio of the mean O.D. values of the Positive and Negative Controls (OD_{PC} and OD_{NC}) is greater than 3.

$$OD_{PC} / OD_{NC} > 3$$

Interpretation

For each sample, calculate the S/P percentage (S/P%):

$$S/P\ \% = \frac{OD_{sample}}{OD_{PC}} \times 100$$

For serum:

Samples presenting a S/P%:

- less than or equal to 50% are considered negative.
- less than 60% and greater than 50% are considered doubtful.
- greater than or equal to 60% are considered positive.

Result	Status
S/P % ≤ 50%	NEGATIVE
50% < S/P % < 60%	DOUBTFUL
S/P % ≥ 60%	POSITIVE

IDvet Innovative Diagnostics

Certified management system

ID Screen® Chlamydophila abortus Indirect Multi-species

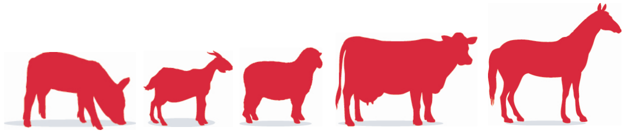

Kit for the detection of antibodies directed against *Chlamydophila abortus* in ruminants, swine and horses.

For *in vitro* use

CHLMS-MS ver 0914 GB

IDvet, 310, rue Louis Pasteur – Grabels - FRANCE
Tel:+ 33 (0)4 67 41 49 33 - Fax: + 33 (0)4 67 45 36 95
www.id-vet.com - E-mail: info@id-vet.com
