# Supplementary material for: Seroprevalence and assessment of public awareness of Brucella spp., Toxoplasma gondii and Chlamydia abortus in small ruminants from selected smallholder commercial farms of Zimbabwe
Source: PLoS One. 2023 Jun 29;18(6):e0287902. doi: 10.1371/journal.pone.0287902 (PMC10310052; doi:10.1371/journal.pone.0287902)
Supplement: S7 File — (PDF) [file pone.0287902.s009.pdf]

General Information

This diagnostic kit is designed to detect antibodies directed against *Brucella abortus* (bovine), *melitensis* (ovine and caprine) and *suis* (pigs).

It can be used in bovine, ovine, caprine and pigs individual serum and plasma or in bovine pools up to 10.

Description and Principle

Wells are coated with purified *Brucella* abortus LPS.

Specimens to be tested and the controls are added to the microwells diluted at 1/20. Anti-*Brucella* antibodies, if present, form an antibody-antigen complex.

A multi-species horseradish peroxidase (HRP) conjugate is added to the microwells. It fixes to the anti-*Brucella* antibodies, forming an antigen-antibody-conjugate-HRP complex.

After washing in order to eliminate the excess conjugate, the Substrate Solution (TMB) is added.

The resulting coloration depends on the quantity of specific antibodies present in the specimen to be tested:

- In the presence of antibodies, a blue solution appears which becomes yellow after addition of the Stop Solution.
- In the absence of antibodies, no coloration appears.

The microplate is read at 450 nm.

Kit Components

**Reagents***
Microplates (12 x 8-well strips) coated with purified *Brucella* LPS
Concentrated Conjugate (10X)
Positive Control
Negative Control
Dilution Buffer 2
Dilution Buffer 3
Wash Concentrate (20X)
Substrate Solution (TMB)
Stop Solution (0.5 M)

**Quantities supplied are indicated on the kit label*

1. The conjugate, the controls and the substrate solution must be stored at 5°C (± 3°C)
2. The other reagents can be stored between +2°C et +26°C.
3. Components bearing the same name (*wash solution, dilution buffers*) can be used for the entire IDvet product range.

Note: If needed, IDvet can supply you with additional volumes of the above components.

Materials required but not provided

1. Mono or multi-channel micropipettors capable of delivering volumes of 10 µl, 100 µl, and 200 µl.
2. Disposable tips.
3. 96-well microplate reader.
4. Distilled or deionized water.
5. Manual or automatic wash system.
6. 96-well pre-dilution microplate.

Precautions

1. Do not pipette by mouth.
2. The substrate solution can be irritating to the skin.
3. The stop solution (0.5 M) may be harmful if swallowed. It may cause sensitisation by skin contact (**R22-43**). Avoid contact with skin (**S24-37**).
4. Do not expose the substrate solution to bright light nor to oxidizing agents.
5. All single-use material used for the assays should be decontaminated by immersion in freshly prepared 5% sodium hypochlorite for minimum 1 hour before elimination, or by autoclaving at 120°C.

Wash Solution Preparation

If necessary, bring the Wash Concentrate **(20X)** to room temperature and mix thoroughly to ensure that the Wash Concentrate is completely solubilised. Prepare the Wash Solution **(1X)** by diluting the Wash Concentrate **(20X)** in distilled/deionised water.

Testing Procedure

Allow all reagents to come to room temperature (21°C ± 5°C) before use. Homogenize all reagents by inversion or Vortex.

1. 1. Add:
   - 190 µl of **Dilution Buffer 2** to all wells.
   - 10 µl of the **Negative Control** to wells A1 and B1.
   - 10 µl of the **Positive Control** to wells C1 and D1.
   - 10 µl of each sample or pools of 10 sera to be tested to the remaining wells.
2. Incubate **45 min*** (± 4 min) at 21°C (± 5°C).

   ** For individual serum samples only, it is also possible to incubate overnight, between **16 and 20 hour**s, at 21°C (± 5°C).*
3. Empty the wells. Wash each well 3 times with approximately 300 µl of the **Wash Solution**. Avoid drying of the wells between washings.
4. Prepare the **Conjugate** by diluting the **Concentrated Conjugate 10X** to 1/10 (short incubation) or to 1/20 (overnight incubation) in **Dilution Buffer 3**.
5. Add 100 µl of the **Conjugate 1X** to each well.
6. Incubate **30 min** ± **3 min at 21°C (± 5°C).**
7. Empty the wells. Wash each well 3 times with approximately 300 µl of the **Wash Solution**.
8. Add 100 µl of the **Substrate Solution** to each well.
9. Incubate **15 min ± 2 min** at 21°C (±5°C) in the dark.
10. Add 100 µl of the Stop Solution to each well in order to stop the reaction.
11. Read and record the O.D. at 450 nm.

Validation

The test is validated if:

✓ the mean value of the Positive Control OD (OD_{PC}) is greater than 0.350.

$$\mathbf{OD_{PC} > 0.350}$$

✓ the ratio of the mean values of the Positive and Negative Controls (OD_{PC} and OD_{NC}) is greater than 3.

$$\mathbf{OD_{PC} / OD_{NC} > 3}$$

Interpretation

For each sample, calculate the S/P percentage (S/P %) as follows using the sample and control values:

$$S/P\% = \frac{OD_{sample} - OD_{NC}}{OD_{PC} - OD_{NC}} \times 100$$

1. For individual serum or plasma samples, short or overnight incubations

Samples with a S/P%:

- less than or equal to 110 % are considered negative.
- greater than 110% and less than 120% are considered doubtful.
- greater than or equal to 120 % are considered positive.

Result	Status
S/P % ≤ 110%	NEGATIVE
110% < S/P % < 120%	DOUBTFUL
S/P % ≥ 120%	POSITIVE

2. For pools of ten bovine sera or plasmas, short incubation

Samples with a S/P%:

- less than or equal to 20 % are considered negative.
- greater than 20 % are considered positive

Result	Status
S/P % ≤ 20%	NEGATIVE
S/P % > 20%	POSITIVE

IDvet
Innovative Diagnostics

Certified management system

ID Screen®
Brucellosis Serum Indirect Multi-species

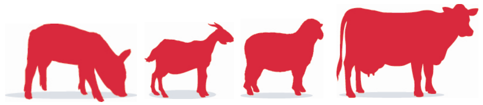

Indirect ELISA for detection of antibodies directed against *Brucella abortus, melitensis* and *suis* in serum and plasma for individual samples or pools of up to 10 bovine sera and plasma

Short and overnight incubations

For *in vitro* use

BRUS-MS ver 1014 GB

IDvet, 310, rue Louis Pasteur – Grabels - FRANCE
Tel:+ 33 (0)4 67 41 49 33 - Fax: + 33 (0)4 67 45 36 95
www.id-vet.com - E-mail: info@id-vet.com
